# Clinical characteristics and predictors of in-hospital mortality in patients with ANCA-associated vasculitis complicated by diffuse alveolar hemorrhage: a retrospective cohort study

**DOI:** 10.3389/fmed.2026.1840399

**Published:** 2026-05-20

**Authors:** Xinran Gao, Xi Wang, Kunyao Yu, Qing Yu, Chengli Que, Guangfa Wang, Jing Ma

**Affiliations:** Department of Respiratory and Critical Care Medicine, Peking University First Hospital, Beijing, China

**Keywords:** ANCA-associated vasculitis, clinical characteristics, diffuse alveolar hemorrhage, in-hospital mortality, predictors

## Abstract

**Background:**

Diffuse alveolar hemorrhage (DAH) is a life-threatening manifestation of ANCA-associated vasculitis (AAV). Data regarding the determinants of in-hospital mortality, particularly the role of specific opportunistic pathogens, remain limited.

**Study design and methods:**

This retrospective cohort study included patients with AAV complicated by DAH admitted to Peking University First Hospital between January 2015 and December 2025. Clinical characteristics, bronchoalveolar lavage fluid (BALF) profiles, microbiological findings, and therapeutic interventions were compared between survivors and non-survivors. Candidate predictors were selected *a priori* based on clinical relevance and published evidence. Least absolute shrinkage and selection operator (LASSO) regression was employed as a complementary screening tool, and multivariable logistic regression was performed to identify independent risk factors for in-hospital mortality.

**Results:**

A total of 58 patients were enrolled, of whom 13 (22.4%) died during hospitalization. Infection-related septic shock was the predominant cause of death, accounting for 76.9% of fatalities. Pulmonary infection occurred in 60.3% of the cohort and was significantly more frequent among non-survivors (92.3% vs. 51.1%; *p* = 0.009). Invasive pulmonary aspergillosis (IPA) was identified in 12.1% of all patients and was markedly more common in non-survivors (46.2% vs. 2.2%; *p* < 0.001). On multivariable analysis, a higher baseline age-adjusted Charlson Comorbidity Index (aCCI) (adjusted OR, 2.78; 95% CI, 1.31–5.87; *p* = 0.007) and the need for early mechanical ventilation (adjusted OR, 14.45; 95% CI, 1.17–179.11; *p* = 0.038) remained independently associated with in-hospital mortality.

**Conclusion:**

Among patients with AAV complicated by DAH, in-hospital mortality remains substantial and is predominantly attributable to infection-related septic shock. Higher baseline aCCI and the need for early mechanical ventilation are independent predictors of in-hospital death. The high incidence of IPA may be associated with mortality and underscores the need for heightened clinical vigilance and proactive screening in this profoundly immunocompromised population.

## 1 Introduction

Anti-neutrophil cytoplasmic antibody (ANCA)-associated vasculitis (AAV) is a group of diseases characterized by small vessel vasculitis, endothelial injury and tissue damage, including granulomatosis with polyangiitis (GPA), microscopic polyangiitis (MPA), and eosinophilic granulomatosis with polyangiitis (EGPA) (1, 2). Pulmonary involvement is a frequent and clinically important manifestation of AAV, presenting as interstitial lung disease, airway involvement, pulmonary nodules, or diffuse alveolar hemorrhage (DAH) (1, 3). Notably, DAH represents the most severe form of pulmonary involvement and is associated with acute respiratory failure, the need for intensive care, and a high risk of short-term mortality (1, 3).

DAH is defined by the presence of red blood cells within the alveolar spaces, which originate from the pulmonary capillaries (4). Common symptoms of DAH include dyspnea, cough, and hemoptysis, with severe cases potentially progressing to respiratory failure (5). GPA and MPA represent the leading etiologies of pulmonary capillaritis resulting in DAH (5), with an incidence of DAH reported as 22% in GPA and 25% in MPA, respectively (6, 7). The probability of developing DAH in EGPA is relatively low, approximately 3–5% (8–10). High-dose glucocorticoids combined with either rituximab or cyclophosphamide constitute the standard remission induction therapy for AAV-associated DAH, as recommended by multiple guidelines (2, 11–13). Despite these advancements, AAV complicated by DAH remains a challenging condition to manage, with in-hospital mortality rates ranging from 11 to 36.9% (5, 14) and one-year mortality rates vary from 11.3 to 50% (15–19). Therefore, early assessment of prognosis and identification of patients at high risk for mortality are critically important.

Several retrospective studies have indicated infection as a risk factor influencing the prognosis of patients with AAV complicated by DAH (17, 20), a susceptibility likely attributable to the heightened risk of infection associated with cyclophosphamide, steroid pulse therapy, plasma exchange, and higher initial doses of glucocorticoids (21). Nonetheless, these studies provided limited information regarding the specific etiological agents that cause infections (15, 17, 20, 22). Consequently, the incidence and prognostic implications of distinct pathogens—such as bacteria, fungi, and cytomegalovirus (CMV)—as well as the impact of infection site on clinical outcomes, remain insufficiently explored in this population.

Prior studies have consistently identified advanced age as an adverse prognostic factor in this population (17, 23, 24). More recently, the age-adjusted Charlson Comorbidity Index (aCCI) has emerged as a superior predictor of mortality compared to age alone, as it integrates both comorbidity burden and patient age (25). Moreover, a retrospective study focusing on AAV patients reported that an aCCI > 5 was associated with an increased risk of death (26). Nevertheless, whether the aCCI provides better prognostic discrimination than individual assessment of age and comorbidities in patients with AAV complicated by DAH has yet to be determined.

The present study aims to identify risk factors associated with in-hospital mortality in patients with AAV complicated by DAH, with the goal of informing clinical management and improving patient outcomes.

## 2 Methods

### 2.1 Study design and population

We retrospectively collected clinical data from patients diagnosed with AAV complicated by DAH who were treated at Peking University First Hospital between January 2015 and December 2025. The study protocol was approved by the Ethics Committee of Peking University First Hospital (Approval No. 2025R0574-0001).

Inclusion criteria were as follows: (1) age ≥18 years; (2) a diagnosis of AAV confirmed according to either the 2012 Chapel Hill Consensus Conference definitions (1) or the 2022 ACR/EULAR classification criteria (27); (3) DAH, defined by the presence of bilateral pulmonary infiltrates on imaging not otherwise explained by alternative causes, such as volume overload or infection, accompanied by at least one of the following: (i) bronchoscopic evidence of DAH, such as progressively hemorrhagic bronchoalveolar lavage fluid (BALF); (ii) hemoptysis; (iii) otherwise unexplained anemia (hemoglobin < 10 g/dL or a decrease in hemoglobin > 1 g/dL) (4, 15); and (4) complete medical records with clearly documented in-hospital outcomes. Exclusion criteria included DAH attributable to causes other than AAV, such as anti-glomerular basement membrane disease, drug-induced vasculitis, IgA vasculitis, connective tissue diseases, cryoglobulinemia, bone marrow transplantation, heart failure or other identifiable etiologies.

### 2.2 Data collection

All baseline data were retrospectively retrieved from the institutional electronic medical record system. Vital signs and laboratory variables—including complete blood counts, renal and liver function tests, coagulation profiles, and arterial blood gas analyses—were obtained from the first measurements recorded within 48 h of hospital admission. BALF analyses were based on findings from the initial bronchoscopy with standardized bronchoalveolar lavage performed during hospitalization.

Comorbidity burden was assessed using the aCCI, which incorporates both the number of comorbid conditions and patient age. The aCCI score was calculated for each patient at hospital admission, with higher scores indicating a greater comorbidity burden (28–30). Baseline disease activity was assessed using the Birmingham Vasculitis Activity Score (BVAS), a validated instrument designed to quantify systemic disease activity in patients with AAV (31). Symptom onset to admission time was defined as the time from the onset of DAH-related symptoms to hospital admission.

Pulmonary infection was defined in accordance with guidelines (32, 33) as the presence of new or progressive pulmonary infiltrate on chest imaging accompanied by clinical features consistent with lower respiratory tract infection. Microbiological evidence from respiratory specimens was considered supportive of the diagnosis. The diagnoses of bloodstream and urinary tract infections were based on clinical guidelines (34, 35). All infectious episodes were reviewed and adjudicated by a committee comprising the principal investigator, a microbiologist and a radiologist. Invasive pulmonary aspergillosis (IPA) was defined according to the updated criteria of the European Organization for Research and Treatment of Cancer/Mycoses Study Group Education and Research Consortium (EORTC/MSGERC) (36)and classified by proven, probable and possible. Probable IPA was defined by the presence of at least 1 host factor, a clinical feature and mycologic evidence. Pulmonary CMV infection was established by detection of positive CMV DNA via quantitative polymerase chain reaction (qPCR) in BALF, with a threshold of > 500 copies/mL (37). To better reflect its baseline prognostic role, mechanical ventilation (MV) in our study was specifically defined as being initiated within 3 days of hospital admission.

### 2.3 Study objective

The objective of this study was to evaluate in-hospital mortality and identify factors associated with death among patients with AAV complicated by DAH.

### 2.4 Statistical analysis

Continuous variables were summarized as mean ± standard deviation or median with interquartile range (IQR) as appropriate for the data distribution, and categorical variables as counts and percentages. Between-group comparisons were performed using the Student’s *t*-test or Wilcoxon rank-sum test for continuous variables, and the chi-square test or Fisher’s exact test for categorical variables. No missing data were found for any of the candidate variables or the variables in the final multivariable model.

To explore factors associated with in-hospital mortality, univariable logistic regression analyses were initially performed, retaining variables with a *p* value < 0.10 as candidate predictors. However, given the limited number of death events relative to the large pool of potential covariates, we applied least absolute shrinkage and selection operator (LASSO) regression as a complementary tool for candidate variable screening. By applying a penalty parameter (*λ*), LASSO shrinks the coefficients of less contributory variables to exactly zero, inherently reducing dimensionality and mitigating overfitting. Candidate variables were evaluated using 10-fold cross-validation based on the one-standard error criterion (λ1se).

Importantly, the LASSO output was utilized strictly as an exploratory cross-validation reference. The final variables entered into the multivariable logistic regression model were determined *a priori* based on robust clinical relevance and established prognostic factors reported in previous literature. By prioritizing clinical plausibility and cross-referencing with univariable (*p* < 0.10) and LASSO results, we constructed a multivariable logistic regression model to estimate adjusted odds ratios (aORs) and identify independent risk factors for in-hospital mortality. Statistical analyses were performed using SPSS version 22.0 (IBM Corp., Armonk, NY, United States) and R software version 4.5.0 (R Foundation for Statistical Computing, Vienna, Austria). All tests were two-sided, and a *p* value <0.05 was considered statistically significant.

## 3 Results

### 3.1 Study population and in-hospital outcomes

**Figure 1:**
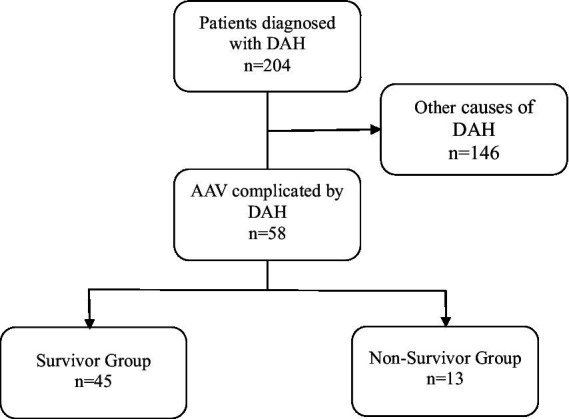
Flow diagram of patient selection. Flowchart illustrating the identification and enrollment of patients with AAV complicated by DAH patients between January 2015 and December 2025. AAV, ANCA-associated vasculitis; DAH, diffuse alveolar hemorrhage.

A total of 204 patients were diagnosed with DAH. Of these, 146 were excluded due to other etiologies. Ultimately, 58 patients with AAV complicated by DAH were included in this retrospective study (Figure 1). Overall, 13 patients (22.4%) died during hospitalization, whereas 45 patients (77.6%) survived to discharge. Patients were stratified into non-survivor and survivor groups for subsequent analyses.

### 3.2 Baseline clinical characteristics

Baseline demographic and clinical characteristics stratified by survival status at discharge are summarized in Table 1. All 58 patients underwent bronchoscopy, revealing progressively hemorrhagic BALF; cytological analysis was available for all. Compared with survivors, non-survivors were significantly older [72.0 (IQR, 66.0–78.0) vs. 58.0 (IQR, 45.5–68.5) years; *p* = 0.002]. Sex distribution and smoking history were comparable between the two groups. The distribution of AAV subtypes did not differ significantly between non-survivors and survivors (GPA vs. MPA; *p* = 0.489). Among non-survivors, 9 patients (69.2%) were newly diagnosed with AAV, compared with 28 patients (62.2%) in the survivors. The interval from first symptom of DAH to hospital admission was significantly shorter in non-survivors than in survivors [10.0 (IQR, 5.0–17.0) vs. 31.0 (IQR, 13.0–91.5) days; *p* = 0.005]. The most common comorbidities included hypertension (50.0%), diabetes mellitus (24.1%) and coronary heart disease (13.8%). The overall comorbidity burden was also greater among non-survivors, as reflected by significantly higher aCCI scores [5.0 (IQR, 4.5–6.5) vs. 3.0 (IQR, 1.0–4.0); *p* < 0.001; Table 1].

**Table 1:** Baseline demographic and clinical characteristics of patients with AAV complicated by DAH at admission.

Variable	All	Non-survivors	Survivors	P Value	Reference Range
Total, *n* (%)	58 (100)	13 (22.4)	45 (77.6)	–	–
Age, years	63.0 (52.7–72.0)	72.0 (66.0–78.0)	58.0 (45.5–68.5)	0.002	–
Female, *n* (%)	24 (41.4)	4 (30.8)	20 (44.4)	0.526	–
Smoking history, *n* (%)	22 (37.9)	7 (53.8)	15 (33.3)	0.208	–
AAV types, *n* (%)					
GPA	14 (24.1)	2 (15.4)	12 (26.7)	0.489	–
MPA	44 (75.9)	11 (84.6)	33 (73.3)		–
Newly diagnosed, *n* (%)	37 (63.8)	9 (69.2)	28 (62.2)	0.751	–
BVAS	22.0 (19.0–25.3)	26.0 (25.0–26.5)	20.0 (19.0–25.0)	0.002	–
Symptom onset to admission time, days	27.5 (9.5–61.3)	10.0 (5.0–17.0)	31.0 (13.0–91.5)	0.005	–
Comorbidities, *n* (%)					
Hypertension	29 (50.0)	9 (69.2)	20 (44.4)	0.207	–
DM	14 (24.1)	7 (53.8)	7 (15.6)	0.009	–
CHD	8 (13.8)	5 (38.5)	3 (6.7)	0.010	–
aCCI	3.0 (2.0–5.0)	5.0 (4.5–6.5)	3.0 (1.0–4.0)	<0.001	–
Symptom, *n* (%)					
Dyspnea	18 (31.0)	9 (69.2)	9 (20.0)	0.002	–
Cough	46 (79.3)	8 (61.5)	38 (84.4)	0.116	–
Hemoptysis	38 (65.5)	7 (53.8)	31 (68.9)	0.339	–
Fever	13 (22.4)	4 (30.8)	9 (20.0)	0.460	–
SpO_2_/FiO_2_ ratio	442.9 (299.1–461.9)	266.7 (158.5–426.2)	452.4 (320.7–466.7)	0.001	≥450.0
Laboratory tests at admission					
WBC, 10^9^/L	10.7 (8.5–13.6)	10.7 (8.0–13.0)	10.6 (8.3–13.9)	0.709	3.5–9.5
HGB, g/L	83.0 (71.8–96.3)	81.0 (72.0–86.0)	83.0 (70.5–98.0)	0.601	115.0–150.0
Neutrophils, 10^9^/L	8.7 (6.0–11.1)	10.3 (7.1–12.0)	8.3 (5.6–11.8)	0.173	1.8–6.3
Lymphocytes, 10^9^/L	0.8 (0.5–1.3)	0.6 (0.2–0.8)	1.0 (0.5–1.5)	0.032	1.1–3.2
NLR	12.4 (5.2–18.7)	18.7 (11.7–60.8)	10.8 (4.7–16.9)	0.013	–
PLT count, 10^9^/L	228.0 (166.0–298.0)	234.0 (146.0–278.5)	228.0 (172.5–308.0)	0.730	125.0–350.0
ALB, g/L	33.0 (28.7–35.5)	28.7 (27.7–32.3)	34.1 (29.5–35.8)	0.009	40.0–55.0
SCR, μmol/L	324.8 (137.6–636.2)	241.5 (151.2–459.5)	391.6 (136.1–674.3)	0.615	44.0–133.0
LDH, IU/L	214.5 (183.3–287.0)	269.0 (204.0–318.5)	204.0 (178.5–268.0)	0.072	100.0–240.0
CRP, mg/L	28.3 (8.7–87.4)	86.4 (35.1–139.5)	19.2 (6.7–52.4)	0.009	0–8.0
D-dimer, mg/L	0.8 (0.4–1.8)	0.6 (0.4–2.0)	0.8 (0.4–1.2)	0.933	≤0.24
BALF cytology, %					
Neutrophils	35.0 (11.8–72.5)	80.0 (64.5–89.6)	22.0 (10.0–44.0)	<0.001	≤3%
Lymphocytes	5.0 (2.0–16.0)	2.0 (1.5–5.5)	6.0 (3.0–19.5)	0.005	7–15%
Macrophages	50.0 (20.8–68.5)	16.0 (8.5–26.0)	60.0 (30.5–73.0)	<0.001	>85%
Hemosiderin-laden macrophages	56.5 (15.0–86.3)	25.0 (6.5–60.5)	63.0 (18.0–87.5)	0.051	0

AAV, ANCA-associated vasculitis; GPA, Granulomatosis with polyangiitis; MPA, Microscopic polyangiitis; BVAS, Birmingham Vasculitis Activity Score; DM, Diabetes mellitus; CHD, Coronary heart disease; aCCI, Age-adjusted Charlson Comorbidity Index; WBC, White blood cells; HGB, Hemoglobin; NLR, Neutrophil/Lymphocyte Ratio; PLT, Platelet count; ALB, Albumin; SCR, Serum creatinine; LDH, Lactate dehydrogenase; CRP, C-reactive protein; BALF, bronchoalveolar lavage fluid. Non-normally distributed data are presented as median (interquartile range).

### 3.3 Clinical presentation, laboratory findings, and BALF analysis

Dyspnea was more frequently observed in non-survivors than in survivors (69.2% vs. 20.0%; *p* = 0.002), whereas the prevalence of cough, hemoptysis, and fever did not differ significantly between groups. Patients presenting with hemoptysis accounted for 65.5% of the cohort. At admission, non-survivors exhibited more severe respiratory impairment, as evidenced by lower peripheral oxygen saturation and SpO_2_/FiO_2_ ratio (both *p* < 0.05). Laboratory analyses revealed that non-survivors had higher blood C-reactive protein (CRP) levels, along with significantly lower serum albumin (ALB) levels (Table 1). There were no statistically significant differences in white blood cell (WBC) count, hemoglobin (HGB), or lactate dehydrogenase (LDH) between groups. The peripheral blood lymphocyte count was lower in the non-survivor group (median 0.6 vs. 1.0 × 10^9^/L; *p* = 0.032), and the neutrophil-to-lymphocyte ratio (NLR) was higher (median 18.7 vs. 10.8; *p* = 0.013). The median serum creatinine (SCR) in non-survivors was numerically lower (241.5 vs. 391.6 μmol/L; *p* = 0.615), but the difference was not statistically significant. All patients underwent bronchoscopy and had BALF cytological results. BALF analysis revealed significantly higher neutrophil proportions in non-survivors (80.0% vs. 22.0%; *p* < 0.001) and lower macrophage proportions in non-survivors compared with survivors, accompanied by a lower proportion of lymphocytes (Table 1).

### 3.4 Infectious complications

Among the 58 patients with AAV complicated by DAH enrolled in this study, 35 (60.3%) developed pulmonary infection during hospitalization, 9 patients (15.5%) were diagnosed with bloodstream infection, and 3 patients (5.2%) with urinary tract infection. The median time from admission to onset of bloodstream infection was 18.0 days (IQR, 13.5–47.0 days). Of these, four cases involved Gram-negative bacilli, three of which were carbapenem-resistant *Acinetobacter baumannii* (CRAB); one patient died before final culture results were available. Five cases involved Gram-positive cocci, with *Enterococcus* accounting for 80% (4/5). All 5 patients who developed bloodstream infection and septic shock died during hospitalization.

Pulmonary infection was common, occurring in 35 of 58 patients (60.3%), and was significantly more frequent among non-survivors than survivors (92.3% vs. 51.1%; *p* = 0.009). Bacterial infection was the predominant etiology (50.0% overall) and was also more common in non-survivors (84.6% vs. 40.0%; *p* = 0.010; Table 2). Among the 29 patients with pulmonary bacterial infection, the most frequently isolated bacterial pathogens were *Pseudomonas aeruginosa* (27.6%) and CRAB (17.2%).

**Table 2:** Infectious complications, in-hospital management and clinical outcomes of patients with AAV complicated by DAH.

Variable	All (*n* = 58)	Non-survivors (*n* = 13)	Survivors (*n* = 45)	*p* value
Infections, *n* (%)				
Any infection	40 (69.0)	13 (100)	27 (60.0)	0.005
Source of infection				
Bloodstream infection	9 (15.5)	6 (46.2)	3 (6.7)	0.001
Urinary tract infection	3 (5.2)	2 (15.4)	1 (2.2)	0.123
Pulmonary infection	35 (60.3)	12 (92.3)	23 (51.1)	0.009
Pulmonary infection pathogens				
Bacterial	29 (50.0)	11 (84.6)	18 (40.0)	0.010
*Aspergillus*	7 (12.1)	6 (46.2)	1 (2.2)	<0.001
*Pneumocystis jirovecii*	3 (5.2)	1 (7.7)	2 (4.4)	0.540
*Mucor*	1 (1.7)	1 (7.7)	0 (0)	0.224
CMV	13 (22.4)	4 (30.8)	9 (20.0)	0.460
SARS-CoV-2	2 (3.4)	0 (0)	2 (4.4)	1.000
Respiratory failure, *n* (%)	33 (56.9)	13 (100)	20 (44.4)	<0.001
ICU, *n* (%)	24 (41.4)	12 (92.3)	12 (26.7)	<0.001
Treatment, *n* (%)				
MV within 3 days	10 (17.2)	8 (61.5)	2 (4.4)	<0.001
In-hospital MV	17 (29.3)	11 (84.6)	6 (13.3)	<0.001
PLEX	32 (55.2)	9 (69.2)	23 (51.1)	0.346
CRRT	19 (32.8)	7 (53.8)	12 (26.7)	0.095
GC bolus	27 (46.6)	7 (53.8)	20 (44.4)	0.753
RTX	12 (20.7)	1 (7.7)	11 (24.4)	0.264
CTX	33 (56.9)	4 (30.8)	29 (64.4)	0.054
Length of hospital stay, days	22.5 (15.0–31.0)	20.0 (15.5–28.0)	24.0 (14.5–31.5)	0.634

CMV, Cytomegalovirus; ICU, Intensive care unit; MV, Mechanical ventilation; PLEX, Plasma exchange; CRRT, Continuous renal replacement therapy; GC bolus, Glucocorticoid pulse therapy; RTX, rituximab; CTX, Cyclophosphamide. Non-normally distributed data are presented as median (interquartile range).

Fungal pathogens were less frequent overall but were markedly enriched among non-survivors. All 7 patients met both the host factors and clinical features of the updated EORTC/MSG 2020 criteria for Probable IPA. Among them, six patients had a BALF galactomannan (GM) > 1.0 and four of them also had a serum GM > 1.0. While the other two patients did not undergo serum GM testing before antifungal therapy. Additionally, the remaining patient was identified via positive BALF metagenomic next-generation sequencing (mNGS) for *Aspergillus fumigatus* (2,715 unique reads) and a positive BALF fungal culture for *Aspergillus fumigatus*; unfortunately, this patient did not undergo BALF GM testing. Among the 6 patients who developed IPA and died, the median time from admission to death was 24.0 days (IQR, 16.0–25.0 days).

*Pneumocystis jirovecii* pneumonia (PJP) was documented in three patients (5.2%), and mucormycosis in one patient (1.7%). None of the three patients received PJP prophylaxis. Viral infections were also observed, including CMV (22.4%) and SARS-CoV-2 (3.4%); however, their frequencies did not differ significantly between non-survivors and survivors (Table 2).

A total of 14 patients had polymicrobial infections involving more than two pathogens. Among these, eight were non-survivors and six were survivors (61.5% vs. 13.3%; *p* = 0.001). Notably, among the seven patients with IPA, six had co-infections with other pathogens: four presented with pulmonary bacterial infections (two with CRAB, two with *Pseudomonas aeruginosa*), and two with CMV.

### 3.5 In-hospital management and clinical course

Admission to the intensive care unit (ICU) was significantly more frequent among non-survivors than survivors (92.3% vs. 26.7%; *p* < 0.001). The proportion of patients who requiring MV at any point during hospitalization was significantly greater in non-survivors than in survivors (84.6% vs. 13.3%; *p* < 0.001), with a pronounced disparity in the need for MV within the first 3 days of admission (61.5% vs. 4.4%; *p* < 0.001; Table 2). The median time from admission to initiation of MV for all patients during hospitalization was 5.0 days (IQR, 1.25–8.75 days).

Continuous renal replacement therapy (CRRT) was required more frequently among non-survivors, whereas CTX use was less frequent in this group. The use of plasma exchange, glucocorticoid pulse therapy, rituximab, and other immunosuppressive treatments did not differ significantly between groups (Table 2).

### 3.6 Risk factors for in-hospital mortality

Among the 13 patients who died during hospitalization, septic shock was the most common cause of death, accounting for 10 cases (76.9%). The median time from admission to onset of septic shock was 22.5 days (IQR, 14.5–41.0 days), and death occurred at a median of 2.5 days (IQR, 1.0–3.25 days) following its onset.

In univariable logistic regression analyses, 20 candidate variables yielded a *p* value < 0.10: age, aCCI, symptom onset to admission time, BVAS score, SpO_2_/FiO_2_ ratio, ALB, CRP, LDH, peripheral blood lymphocyte count, NLR, and BALF cytology, including percentage of neutrophils, hemosiderin-laden macrophages, lymphocytes, and macrophages, as well as pulmonary infection, IPA, pulmonary bacterial infection, MV within 3 days, CRRT, and CTX (Table 3). Of note, to better reflect its baseline prognostic role, we selected MV within 3 days of hospital admission as the variable in our study, instead of in-hospital MV.

**Table 3:** Univariable and multivariable logistic regression analysis of risk factors associated with in-hospital mortality.

Variables	Univariate analysis OR (95% CI)	*p* value	Multivariate analysis aOR (95% CI)	*p* value
Age	1.11 (1.03–1.19)	0.006	–	–
aCCI	2.70 (1.51–4.81)	0.001	2.78 (1.31–5.87)	0.007
Symptom onset to admission time	0.96 (0.93–1.01)	0.083	–	–
BVAS	1.17 (1.03–1.34)	0.021	–	–
SpO2/FiO2 ratio	0.99 (0.99–1.00)	0.004	–	–
ALB	0.83 (0.71–0.98)	0.023	–	–
CRP	1.01 (1.00–1.03)	0.012	–	–
LDH	1.01 (1.00–1.01)	0.099	–	–
Blood lymphocytes	0.27 (0.07–1.07)	0.063	–	–
NLR	1.03 (1.00–1.07)	0.031	–	–
BALF neutrophils	1.07 (1.03–1.10)	<0.001	–	–
BALF lymphocytes	0.81 (0.67–0.99)	0.043	–	–
BALF macrophages	0.94 (0.91–0.98)	0.001	–	–
BALF hemosiderin-laden macrophages	0.98 (0.96–1.00)	0.061	–	–
Pulmonary infection	11.48 (1.38–95.83)	0.024	–	–
IPA	37.71 (3.93–362.23)	0.002	72.81 (0.34–15788.20)	0.118
Pulmonary bacterial infection	8.25 (1.63–41.70)	0.011	–	–
MV within 3 days	34.4 (5.66–209.18)	<0.001	14.45 (1.17–179.11)	0.038
CTX	0.25 (0.07–0.92)	0.038	–	–
CRRT	3.21 (0.90–11.48)	0.073	–	–

OR, odds ratio; aOR Adjusted odds ratio; CI, Confidence interval; aCCI, Age-adjusted Charlson Comorbidity Index; MV, Mechanical ventilation; IPA, Invasive pulmonary aspergillosis; BVAS, Birmingham Vasculitis Activity Score; ALB, albumin; CRP, C-reactive protein; LDH, lactate dehydrogenase; NLR, Neutrophil/Lymphocyte Ratio; BALF, bronchoalveolar lavage fluid; CTX, cyclophosphamide; CRRT, continuous renal replacement therapy. Only variables retained in the final model are displayed in the multivariate column.

**Figure 2:**
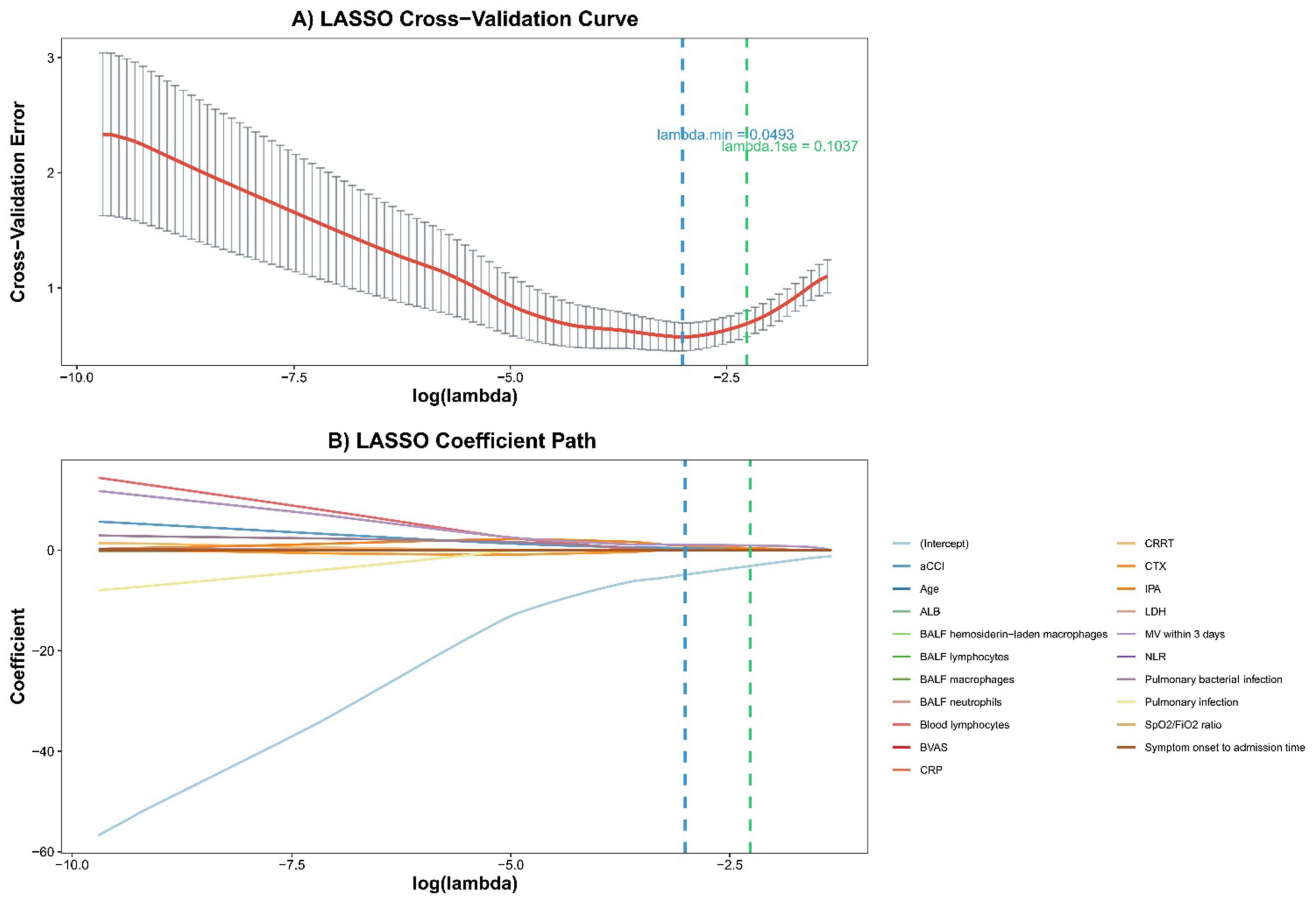
LASSO regression model development and tuning. **(A)** Ten-fold cross-validation curve used to select the optimal *λ*; vertical dashed lines indicate λmin and the more parsimonious λ1se. **(B)** Coefficient profiles of the 20 candidate predictors plotted against log(λ), showing progressive shrinkage and variable selection as the penalty changes.

Given the restricted number of events (in-hospital deaths), simultaneously including all 20 covariates would lead to severe overfitting. To construct a robust parsimonious multivariable model and explicitly prevent clinical collinearity, the selection of final predictors was driven by prior literature and strong clinical relevance, with LASSO regression serving as a reference. With 10-fold cross-validation, the LASSO model identified MV within 3 days, aCCI, and IPA as key predictors at the optimal penalty parameter (*λ* = 0.10, λ₁ₛₑ; Figure 2), which aligned with our clinical reasoning and prior evidence. Consequently, these three variables were selected for the final multivariable model.

In the multivariable logistic regression analysis, aCCI and MV within 3 days, were identified as independent risk factors for in-hospital mortality. Specifically, a higher aCCI was significantly associated with an increased risk of death [adjusted OR (aOR), 2.78; 95% CI, 1.31–5.87; *p* = 0.007]. Furthermore, patients requiring MV within 3 days exhibited a markedly higher likelihood of in-hospital mortality compared to those who did not (aOR, 14.45; 95% CI, 1.17–179.11; *p* = 0.038) (Table 3). The Hosmer-Lemeshow goodness-of-fit test (*χ*^2^ = 5.178, df = 6, *p* = 0.521) demonstrated no significant lack of fit, indicating good calibration of the multivariable logistic regression model. The model achieved a Cox-Snell *R*^2^ of 0.468 and a Nagelkerke *R*^2^ of 0.714, reflecting substantial explanatory power for the outcome.

## 4 Discussion

DAH is a life-threatening manifestation of AAV associated with persistently high mortality (15, 17). However, data regarding prognostic factors for in-hospital mortality in this specific patient population remain limited (5, 14, 38–40). In this retrospective cohort of patients with AAV complicated by DAH, we identified the early requirement for MV and a higher aCCI as independent risk factors for in-hospital mortality.

The requirement for MV initiated within 3 days of admission emerged as an independent predictor of in-hospital mortality in our cohort. This aligns with previous studies indicating that the need for respiratory support is strongly associated with an increased risk of death in AAV-DAH patients (17, 41). In a cohort study by Quartuccio et al. involving 106 patients with AAV complicated by DAH, the need for respiratory support was associated with significantly higher long-term mortality after discharge(HR, 4.58; 95% CI, 1.51–13.87, *p* = 0.007) (17). Accordingly, another study reported that MV was linked to increased mortality (HR, 9.78; 95%CI,1.92–49.7, *p* = 0.006) (41). By specifically evaluating early MV rather than any other point during hospitalization, our analysis captures its prognostic role as a baseline indicator in short-term outcomes. Clinically, the immediate need for MV reflects a fulminant baseline presentation, characterized by catastrophic alveolar hemorrhage and profound early gas exchange impairment. Furthermore, MV may be associated with an elevated risk of secondary complications, including ventilator-associated pneumonia, which can contribute to adverse outcomes (42).

The aCCI was originally developed to quantify long-term mortality risk by integrating both age and chronic comorbid conditions, thereby providing a composite measure of baseline health status (28–30). Previous studies have consistently demonstrated that advanced age is a strong predictor of adverse outcomes in patients with AAV complicated by DAH (17, 20, 24). Elderly patients with AAV are more susceptible to a wide range of disease- and treatment-related complications, including infectious complications and steroid-induced diabetes, contributing to higher mortality rates (20). Beyond age alone, the presence of underlying comorbidities has also been identified as an important determinant of mortality (14). In the present study, we are the first to incorporate the aCCI, which integrates both age and comorbid disease burden, as a prognostic parameter to evaluate the risk of in-hospital mortality in patients with AAV complicated by DAH. Multivariable logistic regression analysis showed that aCCI was an independent predictor of in-hospital death (aOR 2.78; 95% CI, 1.31–5.87). Our results indicate that aCCI, compared with age alone, may facilitate early prediction of mortality risk in patients with AAV complicated by DAH and help optimize timely treatment strategies for high-risk individuals.

Infection represents a leading cause of death in patients with AAV complicated by DAH (17, 20). In previous studies involving this patient population, there was little evidence regarding specification of infection site or microbiological confirmation. We provide a more detailed assessment of opportunistic infections and their prognostic significance. In the present cohort, infection was the predominant cause of in-hospital death, with septic shock accounting for 10 of 13 deaths. All of these events occurred during the terminal stage of the illness prior to death. Notably, among the nine patients with bloodstream infection, five developed septic shock and ultimately died, suggesting that bloodstream infection carries a substantially worse prognosis than pulmonary or urinary tract infection and warrants more intensive surveillance as well as prompt and aggressive management. Given that bloodstream infection and septic shock occurred in the terminal stage, these variables were not included in the multivariable logistic regression analysis. Regarding primary infection sites, pulmonary infection was the most common source in our cohort, followed by bloodstream infection and urinary tract infection, consistent with findings reported by Luo et al. (21)

Prognosis also varied according to the type of pathogen. IPA was identified in seven patients, six of whom died during hospitalization, suggesting that IPA was associated with particularly poor in-hospital outcomes in this cohort. The incidence of IPA in patients with AAV complicated by DAH has not been clearly defined in prior reports. In our cohort, the incidence of IPA was 12.1%, markedly higher than the reported incidence in unselected AAV populations (generally < 2.5%) (21). These findings suggest that patients with AAV complicated by DAH may represent a subgroup at increased risk for IPA compared with patients with other forms of AAV-related pulmonary disease. Furthermore, the development of DAH often signals more active disease and prompts escalation of immunosuppression therapy, which may further increase the likelihood of IPA (11, 36). Our findings underscore the importance of maintaining a high index of suspicion for IPA whenever DAH occurs in patients with AAV. We also suggest that preemptive or prophylactic antifungal therapy may be worth considering in selected patients at highest risk and further investigation in larger prospective studies is needed to confirm. In contrast, PJP infection was less frequently identified in our cohort and was not associated with in-hospital mortality, consistent with previous studies (21). In the present study, the proportion of pulmonary CMV infection was 22.4%, ranking as the second most common pathogen after bacterial infection. In the general AAV population, the incidence of CMV infection is approximately 19–20% (43, 44), comparable to that observed in patients with AAV-associated DAH in our cohort. Therefore, in contrast to IPA, our findings suggest that the presence of DAH in AAV may not be associated with an increased risk of pulmonary CMV infection, nor was pulmonary CMV infection associated with a higher risk of mortality.

In our cohort, bacterial infection accounted for 50% of all identified pathogens, making it the most common etiology, and it was more frequent among non-survivors. Prior studies in patients with AAV have shown that the most common bacteria were *Escherichia coli* and *Staphylococcus aureus* (45, 46). However, among patients with AAV complicated by DAH in the present study, the predominant bacteria of pulmonary infection were *Pseudomonas aeruginosa* (27.6%) and CRAB (17.2%), Gram-negative pathogens or multidrug-resistant organisms. This pattern likely reflects healthcare–associated infections related to ICU admission and mechanical ventilation at the time of DAH, including hospital-acquired and ventilator-associated pneumonia. Therefore, heightened surveillance and prevention of nosocomial infections and multidrug-resistant organisms are warranted in patients with AAV complicated by DAH during hospitalization, particularly in those early receiving MV.

In the pathogenesis of AAV, excessive neutrophil activation and the formation of neutrophil extracellular traps (NETs) play a central role (47, 48). NETs can directly injure small vessels at sites of inflammation, thereby linking the extent of neutrophil accumulation within lung tissue to the severity of local inflammatory damage (47, 48). Consequently, the level of neutrophils in affected organs reflects the severity of tissue damage, as exemplified by the neutrophil proportion in BALF. Cartin-Ceba and colleagues demonstrated that a BALF neutrophil proportion exceeding 30% is an independent risk factor for respiratory failure in patients with AAV complicated by DAH (5). In our cohort, the median BALF neutrophil proportion in the overall population was 35.0% (IQR, 11.8–72.5%), rising to 80.0% (IQR, 64.5–89.6%) among non-survivors, a difference that was statistically significant compared with survivors [22.0%, IQR (10.0–44.0%); *p* < 0.001]. Additionally, the high prevalence of pulmonary infection may also contribute to an elevated BALF neutrophil proportion. These findings underscore the clinical relevance of BALF neutrophilia in AAV with DAH, with higher levels being closely associated with adverse in-hospital outcomes.

Several limitations must be acknowledged. First, the retrospective, single-center design precludes the complete elimination of selection bias, information bias and unmeasured confounders. Second, constrained by the intrinsic rarity of AAV complicated by DAH, our small sample size and limited outcome events inevitably reduced statistical power. This susceptibility to model instability is directly reflected in the wide confidence intervals, most notably for early MV. Consequently, these statistical findings are primarily exploratory and strictly warrant validation in larger multicenter cohorts. Third, our observational approach limits causal inference. Specifically, while IPA was highly clinically relevant, reverse causation remains plausible; critically ill patients with profound immune dysregulation are intrinsically more vulnerable to such opportunistic infections. Finally, by focusing exclusively on short-term in-hospital outcomes, parameters such as long-term survival, renal prognosis, relapse, and post-discharge complications were not captured. Nevertheless, our study provides a rigorous, clinically grounded analysis of infectious profiles and baseline prognostic indicators within a well-defined AAV-DAH population.

## 5 Conclusion

In-hospital mortality among patients with AAV complicated by DAH remains substantial, driven predominantly by infection-related septic shock during the terminal phase. Both a higher baseline aCCI and the requirement for early MV emerged as independent predictors of in-hospital death. Furthermore, a potential association between IPA and in-hospital mortality was identified. These findings indicate that the management of AAV complicated by DAH should extend beyond immunosuppression alone—early risk stratification using aCCI and the requirement for early MV, together with a high index of suspicion and proactive screening for IPA, is essential to alter the fatal trajectory of this complication. Prospective, multicenter cohort studies are warranted to robustly validate these prognostic indicators and to guide optimal clinical interventions.

## Data Availability

The raw data supporting the conclusions of this article will be made available by the authors, without undue reservation.
